# Oncologic Trogocytosis of an Original Stromal Cells Induces Chemoresistance of Ovarian Tumours

**DOI:** 10.1371/journal.pone.0003894

**Published:** 2008-12-16

**Authors:** Arash Rafii, Pejman Mirshahi, Mary Poupot, Anne-Marie Faussat, Anne Simon, Elodie Ducros, Eliane Mery, Bettina Couderc, Raphael Lis, Jerome Capdet, Julie Bergalet, Denis Querleu, Francoise Dagonnet, Jean-Jacques Fournié, Jean-Pierre Marie, Eric Pujade-Lauraine, Gilles Favre, Jeanine Soria, Massoud Mirshahi

**Affiliations:** 1 UMRS 872 INSERM, Université Pierre et Marie Curie-Paris 6 and Université Paris Descartes, Equipe 18, Centre de Recherche des Cordeliers, Paris, France; 2 LFR 44, IFR 31, Institut Claudius Regaud, Toulouse, France; 3 INSERM U563, Department Innovations thérapeutiques et Oncologie moléculaire, Institut Claudius Regaud & Faculté des Sciences Pharmaceutiques, Toulouse, France; 4 INSERM U563, Centre de Physiopathologie de Toulouse Purpan, CHU Purpan, BP3028, Toulouse, France; 5 Department of Genetic Medicine and Obstetrics and Gynecology, WCMC-Qatar, Qatar Foundation, Doha, Qatar; Dresden University of Technology, Germany

## Abstract

**Background:**

The microenvironment plays a major role in the onset and progression of metastasis. Epithelial ovarian cancer (EOC) tends to metastasize to the peritoneal cavity where interactions within the microenvironment might lead to chemoresistance. Mesothelial cells are important actors of the peritoneal homeostasis; we determined their role in the acquisition of chemoresistance of ovarian tumours.

**Methodology/Principal Findings:**

We isolated an original type of stromal cells, referred to as “Hospicells” from ascitis of patients with ovarian carcinosis using limiting dilution. We studied their ability to confer chemoresistance through heterocellular interactions. These stromal cells displayed a new phenotype with positive immunostaining for CD9, CD10, CD29, CD146, CD166 and Multi drug resistance protein. They preferentially interacted with epithelial ovarian cancer cells. This interaction induced chemoresistance to platin and taxans with the implication of multi-drug resistance proteins. This contact enabled EOC cells to capture patches of the Hospicells membrane through oncologic trogocytosis, therefore acquiring their functional P-gp proteins and thus developing chemoresistance. Presence of Hospicells on ovarian cancer tissue micro-array from patients with neo-adjuvant chemotherapy was also significantly associated to chemoresistance.

**Conclusions/Significance:**

This is the first report of trogocytosis occurring between a cancer cell and an original type of stromal cell. This interaction induced autonomous acquisition of chemoresistance. The presence of stromal cells within patient's tumour might be predictive of chemoresistance. The specific interaction between cancer cells and stromal cells might be targeted during chemotherapy.

## Introduction

Epithelial Ovarian carcinoma (EOC) is the sixth most common malignancy in woman and the leading cause of death from gynaecological cancer in the world [1]. EOC has a predisposition to metastatic involvement of the peritoneal cavity [2], [3]. Late stage EOC is characterized by widespread peritoneal dissemination, ascites and a high rate of mortality with an overall survival ranging from 20 to 30% at 5 years after surgery depending the studies [4].

Platinum associated to taxans chemotherapy, is a standard treatment for ovarian cancer, and has achieved a high response rate. The development of drug-resistant cancer cells exhibiting the multidrug resistance phenotype is one of the major limitation of efficacy that has been illustrated in the literature for platinum or taxane chemotherapy [4], [5].

A growing amount of studies are underlying the role of the microenvironnement in EOC development. Indeed the peritoneal sheath is composed by the mesothelium, a simple squamoid epithelium lining also the pleural and pericardial cavities [6]. The mesothelial cells play a major role in important physiologic functions such as dialysis, localization of infections and formation of abdominal adherences [7], [8]. Their role in the dissemination of gastric, pancreatic and ovarian carcinoma has also been reported [9]. Several authors have demonstrated that ovarian cancer cells were able to attach to peritoneal mesothelial cells through activation of CD44 or beta-1 integrin [10]–[12]. Furthermore Burleson et al. have demonstrated that ovarian carcinoma ascitis spheroids (e.g. multi-cellular aggregates of ovarian cancer cells) were able to adhere to live but non-fixed human mesothelial cells [13], [14]. Recently analysis of surgical specimens suggested that mesothelial cells may nurture peritoneal metastases through the production of growth factors such as vascular endothelial growth factor (VEGF) and fibroblast growth factor 2 (FGF2). This confirmed the findings of Wilson who demonstrated that mesothelial cells from ovarian cancer patients were able to stimulate the clonogenic growth of ovarian tumor cells [15], [16]. Mesothelial cells undergo morphologic changes in cancerous ascites, cirrhotic ascites and peritonitis. Indeed, before tumoral peritoneal implantation, mesothelial cells have been reported to become hemispheric and exfoliate into the peritoneal cavity [17]. In particular it has been demonstrated that the morphology of mesothelial cells obtained from patients with EOC is different from those obtained from non–cancer-bearing individuals. The mesothelial cells from patients with EOC displayed also a growth advantage suggesting an activated state [9]. Finally by using DNA microarray analysis, Wang et al. demonstrated that peritoneal and subjacent stroma of patients with EOC were transcriptionaly different from that of patients with benign ovarian disorders [9]. The genes that were differentially regulated were implicated in different important processes such as cell adherence, growth and invasion.

As illustrated above there is a strong support that the interaction between EOC and its surrounding microenvironment is a primordial step in the development and progression of metastatic EOC. Evidences in the literature are suggesting a “cross-talk” between cancer cells and peritoneal stromal cells. Beyond the development of a peritoneal disease the occurrence of chemoresistant tumoral clones during chemotherapy remains a major issue in ovarian cancer.

It has not yet been clearly illustrated whether mesothelial cells of the peritoneum play an active role in the phenomenon of chemoresistance. Therefore we hypothesized that in the peritoneal cavity mesothelial cells could act as privileged partners of ovarian cancer cells. We isolated non-previously described stromal cells closely associated to cancer cells. These cells conferred chemoresistance to ovarian cancer cell. The molecular mechanism underlying chemoresistance acquisition is described.

## Results

### Isolation and characterization of mesothelial cells

**Figure 1:**
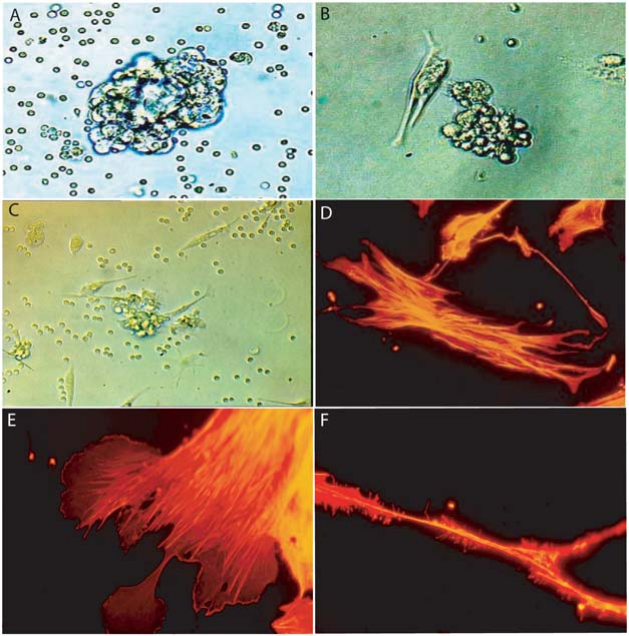
Isolation of Hospicells. A. Cellular aggregates from ascitic fluid of patients with ovarian carcinosis in suspension (×40). B. Cellular aggregates on culture plates (×40). C. Gradual disaggregation of strongly interacting stromal cells (“Hospicells”) (thick arrow) and cancer cells (thin arrow) at day 10 days of culture (×20). D. Fluorescence microscopy (actin immunostaining) showing the “hammock-like” active cytoskeleton of freshly isolated Hospicells (×60). E. Lamellipodal formation at the extremity of the Hospicells containing active actin fibers. F. Details of a Hospicell's fillopode demonstrating numerous “comb-like filaments”.

Mesothelial cells are known to exfoliate at the beginning of the metastatic process. This suggests that ovarian cancer-specific mesothelial cells could interact with epithelial ovarian cancer cell aggregates (EOCA). We thus isolated EOCA from ascitic fluid from non-previously treated patients with stage IIIc ovarian cancer (Figure 1A, B). Demographic characteristics of these patients are described in Table 1. The EOCA, which were initially strongly aggregated, gradually dissociated as from 10 to 15 days of culture (Figure 1C), enabling us to clone the constitutive mesothelial cells (Hospicells) by limiting dilution and enrichment. As demonstrated in Figure 1C only some stromal cells had the ability to interact with the ovarian cancer aggregates.

**Table 1:** Demographic characteristics of the initial patients included in the study.

Patients	Age	Histologic Type	Grade	FIGO stage
1	58	Serous adenocarcinoma	3	IIIc
2	62	Serous adenocarcinoma	3	IIIc
3	67	Serous adenocarcinoma	2	IIIc
4	59	Papillary adenocarcinoma	3	IV
5	61	Papillary adenocarcinoma	2	IIIc

Cloned Hospicells are large cells with a unique morphology. They have long, thin pseudopods forming a kind of net (Figure 1D) and an active cytoskeleton (Figure 1E, F) able to capture bound cells.

**Figure 2:**
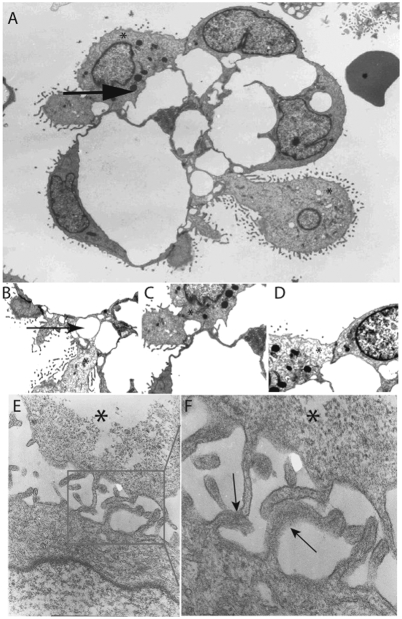
Electronic microscopy analysis of ovarian cancer aggregates and primocultures of Hospicells and OVCAR3. A. Electronic microscopy sections showing EOC cell and Hospicells aggregates displaying numerous interactions between cancer cells (thin arrow) and Hospicells (thick arrow and (*)). B, C and D. Details of different interactions: through thin pseudopods (B and C), or through large membranous contact (D). Hospicells are marked with an (*) sign. E. Co-cultures of freshly isolated Hospicells (*) and OVCAR3 displaying the network of Hospicells' pseudopods enhancing contact with EOC cells. F. Cell membrane fusion (arrows) between Hospicells (*) and cancer cells pseudopods in co-cultures.

Further analysis using electron microscopy studies have shown that EOCA are aggregates of EOC cells and Hospicells (Figure 2A). The Hospicells interact with the EOC cells via their pseudopods (Figure 2B), by membrane contact over a large surface area (Figure 2C), and through tight junctions such as desmosomes (Figure 2D). When cultured with EOC cells, Hospicells' cytoplasm rapidly expanded into several short pseudopods (Figure 2E), producing small membrane fusion domains in the contact zones (Figure 2F).

### Phenotypic Characterization of the Hospicells

According to immunochemistry studies (detailed in Table 2), these original cells isolated for their specific adhesion to ovarian cancer cells among the aggregates, did not express lineage-specific cell surface markers such as cytokeratin and EMA (specific for epithelial cell lines), vimentin (specific for mesenchymal cell lines), CD45 (specific for lymphoid tissue), CD20 (specific for B lymphocytes), CD3 (specific for T lymphocytes), CD68 (specific for macrophages and histiocytes), CD34 (specific for stem cells), S100 protein (specific for melanocytes), and myeloperoxidase (specific for granulocytic lineage).

**Table 2:** Molecular characterization of Hospicells.

Hospicells' reactivity	Negative	Positive
**Antigen specificity**		
**Epithelial**	EMA, KL1,	CD9, CD29
	AE1, AE3,	
	CK5, CK6, CK7, CK8, CK18, CK19, CK20, E-cadherin, EGFR	
**Hematopoietic**	CD45, CD3, CD4, CD11a, CD15, CD16, CD19, CD23, CD26, CD34, CD68, CD41, LC, DBB42, DBA44, CD15, CD30, CD49d, VLA4, CD51, CD56, CD66, CD69, CD126, CD133	CD10, MHC1
**Mesenchymal**	CD133, CD99, vimentine, CALB2, HBME1 (mesothelial cell surface protein)	CD166
**Endothelial, lymphatic**	D2-40, CD31, CD71, CD106	CD146
**Neuro-epithelial**	NSE, Chromogranin, CD57, synaptophysin (p38), S100	
**Myo-epithelial**	alpha-Smooth actin	
**Ubiquitous**	CD44, CD47	
**Embryonic Stem-cells**	SSEA-1, SSEA-4	

### Specific adhesion of the Hospicells

**Figure 3:**
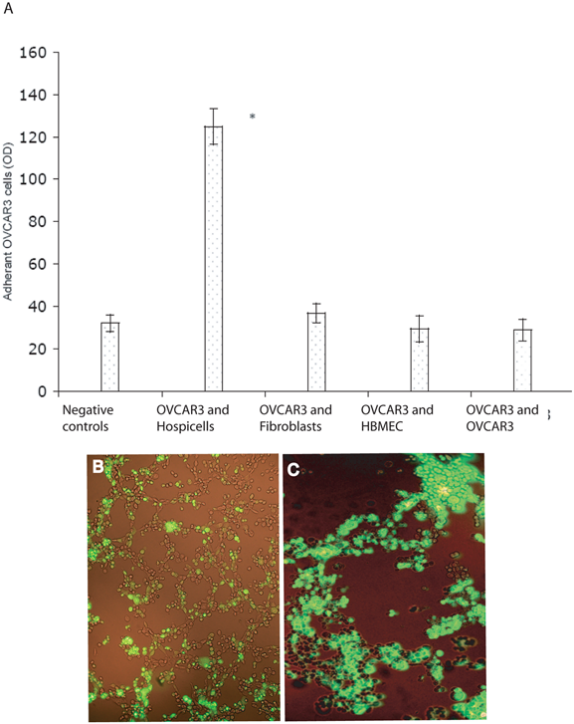
Specific interaction between OVCAR3 cells and Hospicells. A. Adhesion of eGFP-OVCAR3 to Hospicells compared to that of fibroblasts and HBMEC (Human Bone Marrow Endothelial Cells). B and C. Hospicells were seeded on Matrigel coated culture plates allowing them to form a network. eGFP-OVCAR3 cells were then added. eGFP-OVCAR3 cells developed on the Hospicells network.

The isolation of the Hospicells among the EOCA raised the question about the specificity of the interaction between these cells and ovarian cancer cells. Unlike cell-lines such as human bone marrow endothelial cells (HBMEC) or fibroblasts, OVCAR3 cells adhered strongly and specifically to freshly isolated Hospicells (Figure 3A). It is noteworthy that OVCAR3 cells did not display a significative adhesion profile to other OVCAR3 cells, increasing the significance of Hospicells' presence among the ECOA.

Moreover when seeded on Matrigel pre-coated plates containing Hospicells, eGFP-OVCAR3 were mainly growing on the network previously formed by the stromal cells (Figure 3B and C).

### Chemoresistance assays

**Figure 4:**
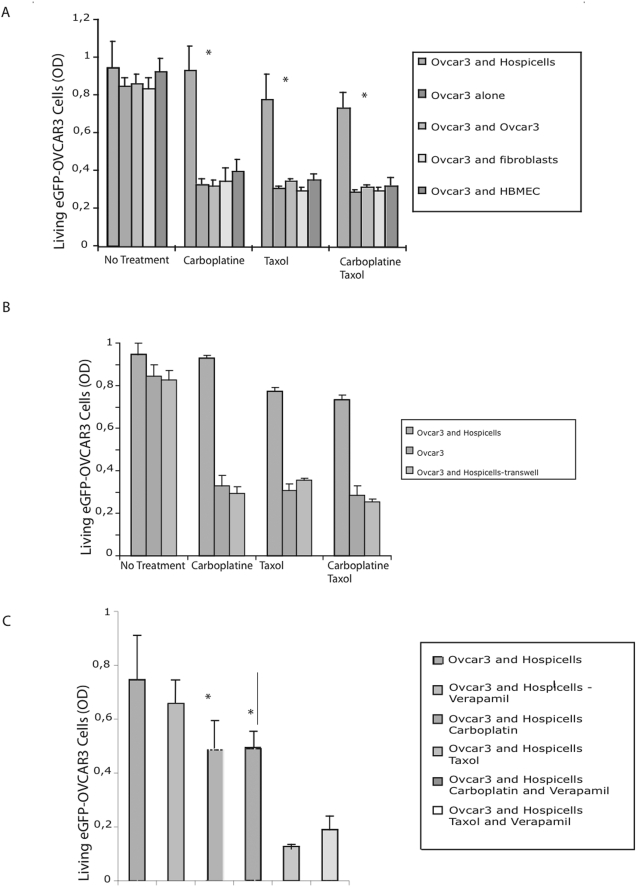
Chemoresistance induced by Hospicells. A. Induced chemoresistance when Hospicells co-cultured with OVCAR3 cells over-expressing e-GFP are treated with 22.2 µM carboplatin and/or 1.4 µM paclitaxel (* p>0.05). Fibroblasts, HBMEC and OVCAR3 cells were used as controls. B. Same assay using a transwell co-culture system. C. Reversal of chemoresistance by treating the co-culture by 50 µM verapamil. Cell density is expressed as the optical density (OD), means with standard deviations (6 replicates were used per experiments. The experiments were performed 3 times).

**Figure 5:**
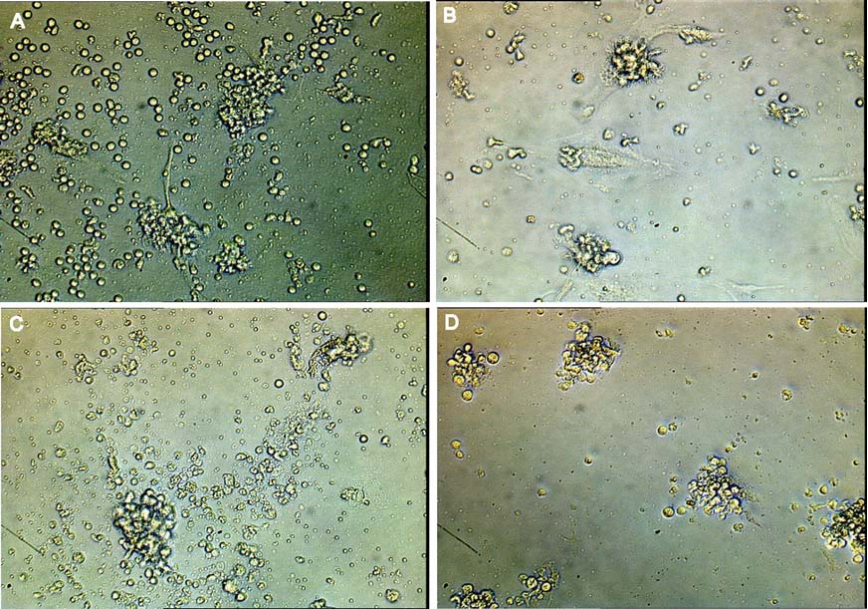
Effect of chemotherapy on freshly isolated ECOA. ECOA were isolated and cultured on RPMI media supplemented by calf fetal serum (10%) and antibiotics. Carboplatin and paclitaxel treatment were performed using the same conditions as in vitro. The effect of chemotherapy was assessed by conventional microscopy. A and C. ECOA freshly isolated in culture before treatment. B. Day 3 after treatment by carboplatin (22.2 µM). D. Day 3 after treatment by paclitaxel (1.4 µM).

We were able to demonstrate that co-culture of the two cellular sub-types gave a significant proliferative advantage to the cancer cells. The proliferative advantage was also demonstrated in an *in-vivo* model of peritoneal carcinosis (Supplementary Figure S1). To investigate their potential role in chemoresistance we conducted a chemoresistance assay. Firstly we demonstrated as displayed in Figure 4A that the co-culture of Hospicells and OVCAR3 cells induced a chemo-resistant profile with differences of 2.2 to 2.5 fold between the co-culture and the eGFP-OVCAR3 cells alone depending the chemotherapeutic agents used, (p<0.05). This effect was Hospicells specific, HBMEC and fibroblasts being unable to confer chemoresistance, and was mediated by direct cell contact as shown by transwell experiments (Figure 4B). We then investigated the role of Multi Drug Resistance (MDR) proteins by conducting a chemoresistance assay in the presence of the selective drug-efflux pump inhibitor, Verapamil [18], [19]. Verapamil abrogated the acquisition of chemoresistance (Figure 4C), thus establishing the involvement of MDR proteins. The effect of Hospicells among ECOA was assessed by treating freshly isolated ECOA form non-previously treated patients with ovarian peritoneal carcinosis. As displayed in Figure 5, when treated by carboplatin or paclitaxel ECOA managed to survive compared to isolated cancer cells.

### Membrane exchange

Intercellular communication by mechanisms such as peptide transfer through gap-junctions, ligand-receptor interactions and membrane protein transfer can lead to the acquisition of complex phenotypes [20]. Most lymphoid cells actively capture membrane pieces from antigen-presenting cells to which they are bound during antigen recognition or from target cells such as cancer cells [21]–[23]. This phenomenon, called trogocytosis, can also occur between certain cancer cells in the absence of an exogenous stimulus.

**Figure 6:**
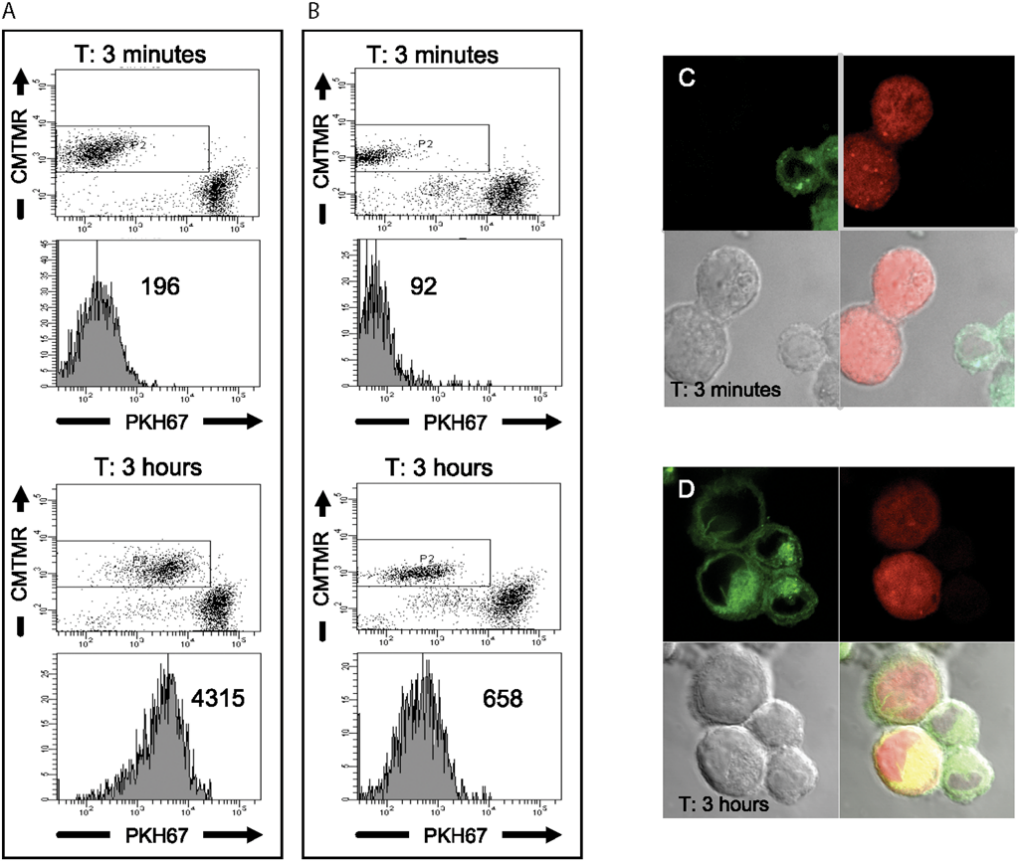
Intercellular transfer or “oncologic trogocytosis”. A and B. Flow cytometry of OVCAR3 cells or Hospicells co-cultured with Cell Tracker™ Orange CMTMR-labelled cells for 3 minutes or 3 hours. A. The Hospicells were labelled with the membranous lipophilic dye PKH67B. The OVCAR3 cells were labelled with PKH67. The value given in the plot is the mean PKH67 fluorescence intensity. C. Confocal microscopy of PKH67-stained hospicells (green) and CMTMR-labelled OVCAR3 cells (red) after 3 minutes incubation. D. Uptake of PKH67 by OVCAR3 cells is observed at 3 hours indicating membrane transfer without cytoplasmic transfer.

To test this hypothesis, we adapted the trogocytosis assay to the capture of Hospicell membranes by co-incubated OVCAR3 cells. Hospicells' membrane were stained with a lipophylic fluorescent dye PKH67; after three hours of incubation, OVCAR3 cells acquired strong green fluorescence as a result of the capture of labelled Hospicells' membrane fragments (an increase in mean fluorescence intensity (mfi) from 196 to 4315, Figure 6A). When the OVCAR3 cells were labelled instead, the Hospicells also acquired green fluroescence, but to a lesser extent (an increase in mfi from 92 to 658, Figure 6B). Massive Hospicells' membrane acquisition by bound OVCAR3 cells was confirmed by confocal microscopy (Figure 6C and D). We have thus demonstrated that EOC cells can capture Hospicells' fragments when cells are in direct contact as in EOCA. As shown on the FACS and confocal analysis the acquisition of PKH67 was not due to the diffusion of the fluorescent dye in the culture media.

### MDR proteins and membrane transfer

**Figure 7:**
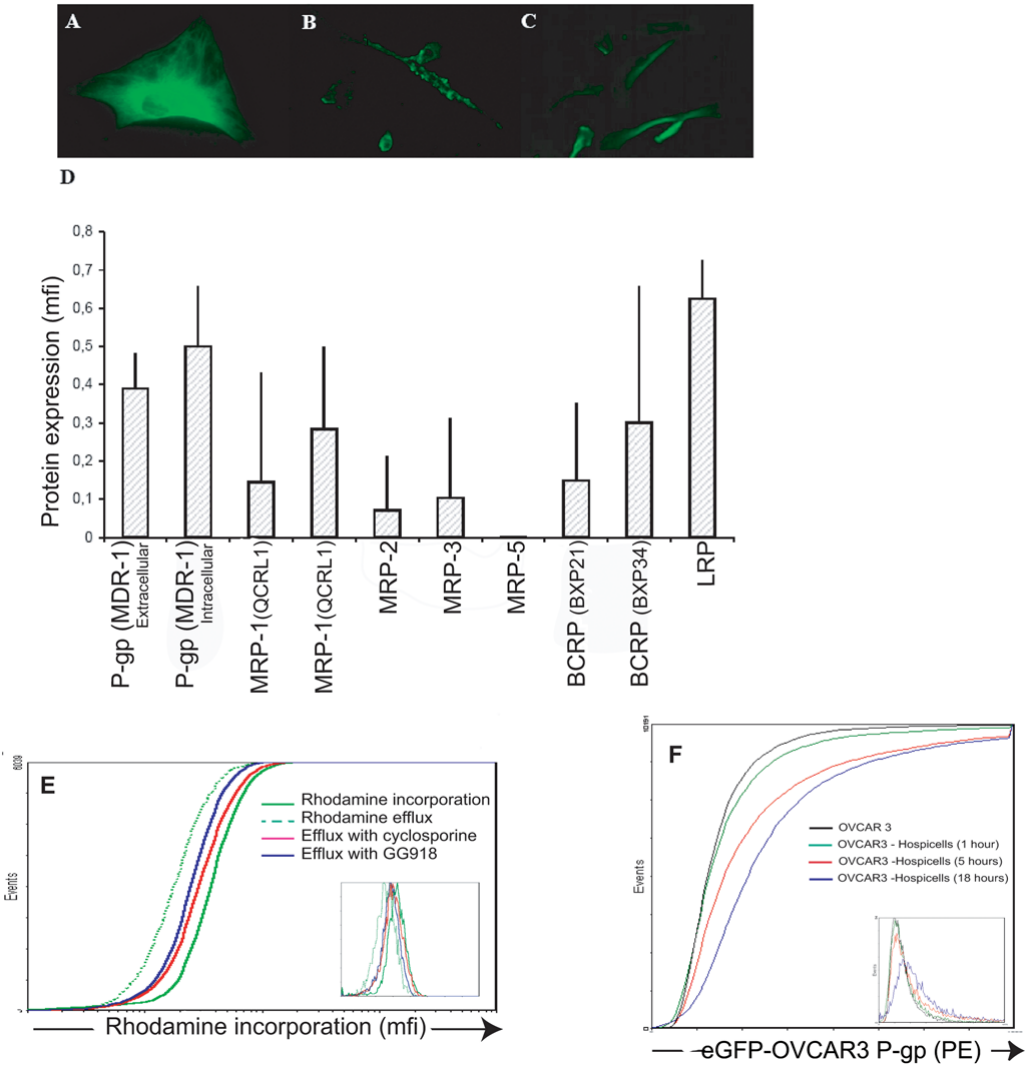
Expression and functionality of MDR proteins in hospicells. A, B and C. Immunofluorescence staining of P- Glycoprotein (P-gp) (A), Lung Resistance Protein (LRP) (B), and Breast Cancer Resistance Protein (BCRP) (C) in Hospicells isolated from ascitic fluid from 5 patients (Multidrug Resistance protein (MRP)). D. Expression of MDR proteins (mean fluorescence intensity (mfi)) assessed by FACS (mfi (+SD)). E. Flow cytometry of functionality of P-gp1 expressed by Hospicells: control, e.g. rhodamine incorporation (green curve – mfi = 22), rhodamine excretion (dashed curve - mfi = 35); inhibition of excretion by cyclosporine (red curve - mfi = 25) and GG918 (blue curve- mfi 26). F. Intercellular transfer of labelled P-gp on co-culture of hospicells and OVCAR3 cells for 1 hour, 5 hours and 18 hours.

Transmembrane drug transporters that extrude anti-tumor agents from the cells have an important role in the multi drug resistance mechanisms (MDR). Indeed over-expression of the ATP binding cassette transporters such as ABCB1 (MDR1) has been directly implicated in resistance to a broad spectrum of chemotherapeutic agents in vitro including paclitaxel and in some studies carboplatin [24], [25]. As co-culture with Hospicells seemed to induce chemoresistance of the OVCAR3 cells we examined whether the Hospicells were expressing MDR proteins. As displayed in Figures 7-A to C, the presence of P-gp, LRP, MRP and BCRP proteins was assessed using immunofluorescence technique. The levels of MDR expression was also assessed using FACS using different Hospicells from 5 different patients. Figure 7-D displays the pattern of expression of the MDR proteins on the Hospicells isolated initially in this study. Several MDR proteins were mostly expressed on the Hospicells including the P-gp family and the Lung resistance Protein (LRP). We completed this observation by determining the functionality of the MDR proteins expressed by Hospicells by flow cytometry using different probes. Figure 7-E shows the result for P-gp using rhodamine as a probe, the ability of the cells to excrete rhodamine is observed and we also demonstrated that this ability was inhibited by the use of cyclosporine.

So far we displayed the acquisition of chemoresistance through intercellular interactions between the Hospicells and the ovarian cancer cells. We also demonstrated that Hospicells expresses functional MDR proteins.

Having demonstrated that OVCAR3 cells and Hospicells can exchange membranes, thus enabling OVCAR3 cells to acquire functional MDR proteins, we performed the control experiment of co-culturing OVCAR3 cells with cloned Hospicells that expressing a low level of MDR proteins. This did not lead to acquisition of MDR proteins by OVCAR3 cells (Figure S2). However after 5 hours of co-culturing OVCAR3 cells with high P-gp expressing Hospicells, 10,2% of the OVCAR3 cell population was found to have acquired a P-gp^+^ phenotype. This expression remained stable after 18 hours of co-culture (Figure 7F).

### Clinical correlation

**Figure 8:**
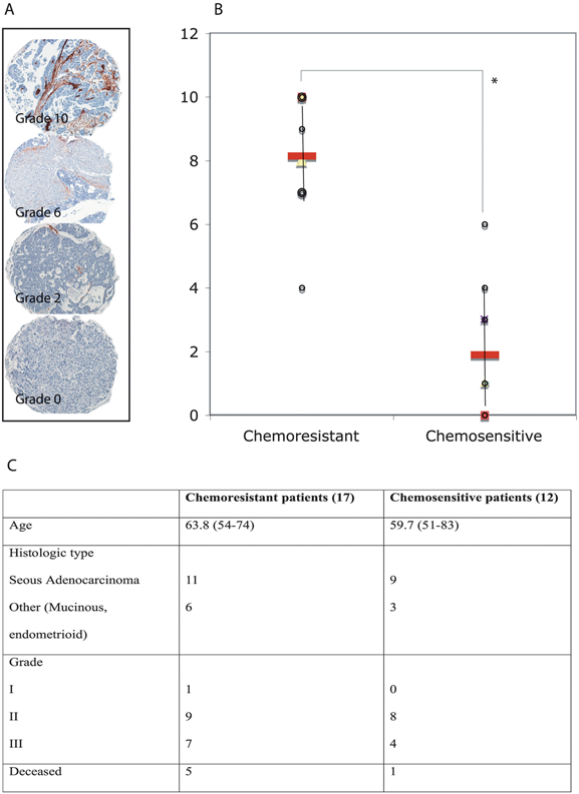
Clinical relevance of the presence of Hospicells among ovarian cancer tumors. Tissue Micro-arrays were prepared from tumors originating from 29 patients who underwent a neo-adjuvant chemotherapy. A. Different densities of Hospicells as represented by CD10 staining. Morphological controls were used to rule out the staining of endothelial cells. B. Samples from chemoresistant patients had significantly higher density of Hospicells compared to chemosensitive patients (* p<0.05). Three different spots were used to characterize the Hospicells' density for each patient.

A total of 29 ovarian carcinomas were assessed histopathologically for a Hospicells infiltrate within the tumor using a CD10 staining. Figure 8A displays the different level of Hospicells staining. As displayed in Figure 8B TMA from chemoresistant patients had significantly higher density of Hospicells compared to chemosensitive patients. Demographic characteristics of the patients included in the study are displayed in Figure 8C.

## Discussion

There are many evidences that preferential invasion of the peritoneum by ovarian cancer is not only due to anatomic proximity but also to an array of molecular signals secreted by cancer cells and stromal cells predisposing the peritoneum to invasion. Indeed the role of molecule-dependant cell-cell interactions (integrin β-1, CD44), secreted factors (interleukin 6 and 8, VEGF, FGF2, TGF-β) and matrix metalloproteinases, has been illustrated in several reports [26]–[30]. However one of the most important partners of cancer cells in the peritoneum are mesothelial cells of the peritoneal surface. As described in the introduction, few studies are addressing the role of these cells in ovarian cancer. In our study, in order to avoid the bias associated to the heterogeneity of peritoneal sampling, we isolated cancer cell aggregates. The hypothesis of our work was that stromal cells among these aggregates would display a high specificity regarding their interactions with ovarian cancer cells.

In this study we have isolated stromal cells not previously described that act as host cells. These cells represent a new subset of stromal cells that do not express any of the common markers for other cell types. The co-expression of the cell surface markers CD9, CD10, CD29, CD146 and CD 166 has not been linked to any specific lineage. However they have already been described as part of the complex phenotype of the mesenchymal stem cells in particular the adipose tissue derived stromal cells also called stromal vascular fraction [31]. The Hospicells might therefore represent a differentiated stromal subset of the mesenchymal stem cells. Noteworthy, Karnoub et al. have recently provided evidence that bone-marrow-derived human mesenchymal stem cells caused, through paracrine mechanism, weakly metastatic human breast carcinoma cells to increase their metastatic potency [32]. Interestingly, this enhanced metastatic ability was reversible and dependent on intercellular contact and CCL5 signalling through the chemokine receptor CCR5, the most potent mesenchymal cells in their study were CD10^+^ cells. Recently, Kaplan et al. have described the role of bone marrow VEGF-R1 progenitors as pre-metastatic niches in a model of lung cancer [33]. Following modification of the extracellular environment in host organs by secreted tumoral factors, bone marrow progenitors were able to constitute a pre-metastatic niche and subsequently attract cancer cells and facilitate the growth of metastasis [34]. The pre-metastatic niche was constituted from CD34-positive progenitors, however the exact composition of the niche is not illustrated in their study. We can hypothesize that the pre-metastatic niche is a complex microenvironment such as the stem cell niche. Different cellular subtypes might then have different roles in promoting metastasis. Our cell type may act as a “feeder” cell in the initiation of the metastatic process. Moreover stem cells from a variety of tissues express high levels of ABC transporting proteins that play a role in cytoprotection through the excretion of genotoxic and xenobiotic compounds out of the cells [35]. These latter properties might therefore create a “drug-free” niche that will be more suitable to cancer cells development.

Hospicells also displayed positivity for other markers. CD9 is a member of the tetraspanin family. These proteins mediate signal transduction events and play a role in the regulation of cell development, activation, growth and motility and intercellular interactions. CD9 in particular plays a role in fusion between the spermatozoide and the ovocyte. In addition, it promotes muscle cell fusion and support myotube maintenance [36]–[38]. CD9 down regulation on ovarian cancer cells seem to be associated to higher histological grade and metastatic progression. The role of CD9 expression on stromal cells in ovarian cancer has not been established [39]. CD10 is a neutral endopeptidase (NEP) also known as common acute lymphoblastic leukemia antigen (CALLA). It is a zinc-dependent metalloprotease enzyme that degrades a number of small-secreted peptides. Associations have been observed between CD10 overexpression on cancer or stromal cells and various types of cancer such as ovarian cancer or advanced melanoma [40], [41]. CD10 was specifically expressed in the stroma of borderline and malignant ovarian tumors, but not in adenomas. Furthermore, stromal CD10 was downregulated as the histological grade advanced. These results suggest that CD10 may play a role in the regulation of neoplastic transformation and tumor differentiation in epithelial ovarian carcinomas. Similarly the presences of CD10 positive cells have not been assessed in ovarian cancer so far. CD166 or ALCAM. Decreased/lost ALCAM membrane expression is a marker of poorer outcome in epithelial ovarian cancer. CD166 has recently been described as a marker of the bone marrow stromal cells capable of supporting hematopoiesis [42], [43]. Its specific role in ovarian cancer has not been studied. CD146, the melanoma cell adhesion molecule (M-CAM), is a cell adhesion molecule currently used as a marker for endothelial cell lineage. Its function is still poorly understood, but evidence points to it being part of the endothelial junction associated with the actin cytoskeleton. It is expressed, activated human T cells, endothelial progenitors such as angioblasts and mesenchymal stem cells, and strongly expressed on blood vessel endothelium and smooth muscle [44]. In Ovarian cancer, M-CAM is a marker of early relapse and poorer outcome in EOC. In particular, M-CAM expression identifies a subgroup of front-line therapy-responding patients who undergo dramatic relapses [45]. Mesenchymal stem cells expressing this marker seem to be associated with perivascular cells surrounding the blood vessels [46].

The morphology of these cells is perfectly adapted to their role as they have many pseudopodia that allow large contacts with cancer cells. As we observed a specific and intense adhesion between the two cell types, we hypothesized that the Hospicells would play a role in ovarian cancer cell physiology.

Intercellular communication can lead to acquisition of complex phenotypes. Several mechanisms can occur during inter-cellular communication such as molecules transferring through gap-junctions, coupling through ligand receptor interactions and finally transfer of membranes proteins leading to new-cell surface proteins conferring new properties to the accepting cells [20], [36]–[38]. Levchenko et al. have described intercellular transfer of functional P-glycoprotein among several tumor cell lines [47]. This inter-cellular protein transfer was able to induce chemoresistance in previously sensitive cells. As one of the major challenges in ovarian cancer treatment remains chemo-resistance, we investigated the role of Hospicells in the survival of cancer cells treated with different chemotherapeutic agents. We were able to describe the ability of a subset of normal cells to confer chemo-resistance to ovarian cancer cells through inter-cellular contact. Two findings were important in the chemoresistance assay performed: the specificity of the Hospicells to confer chemoresistance compared to other cell types used as controls and the importance of the inter-cellular adherence. All together these two findings suggest that Hospicells can be a crucial key in the chemoresistance phenomenon, and their presence might have consequences on patients' prognoses. Moreover the necessity of the contact between the two cells rules out the role of secreted factors and confirms the role of the peritoneal cells ”per se” in the occurrence of ovarian tumor chemoresistance.

Several mechanisms can be responsible for chemoresistance acquisition. The role of the MDR proteins in particular in ovarian carcinoma has been widely illustrated in the literature [24], [25]. We were able to demonstrate the presence of MDR proteins on Hospicells from several different patients with a variable expression between the patients. However the expression of the functional P-gp and LRP was constant. The ability of Verapamil to revert the chemoresistance in the co-culture also confirmed the role of the MDR proteins. The MDR proteins of interest in this study were P-glycoprotein (P-gp, MDR1, ABC B1), a membrane glycoprotein. Its resistance phenotype is similar and includes anthracyclines (doxorubicin), vinca-alkaloids, epidophyloxins and taxanes and it has also been related to carboplatin resistance [18], [19], [24], [25].

Several mechanisms can underlie the occurrence of chemoresistance after inter-cellular contact. However the specificity, the crucial role of intercellular interaction, and the morphology of these interactions displayed by electronic microscopic analysis suggested the occurrence of “oncologic synapses” as compared to “immunological synapses”. Indeed lymphoid cells (effectors) will initiate their interactions with surveyed cells (targets) by setting synapses that enable their surface receptors to interact with ligand, to concentrate activation signals, and finally to deliver effectors functions [48], [49]. The lymphocyte cell surface in this contact area makes small bridges with the target cell and captures patches of its membrane on its own cell surface, an active process referred to as trogocytosis [48]. Using the same experimental designs, we describe “trogocytosis” between a cancer cell (as an effectors) and a normal host cell (as a target) with a transfer of MDR proteins from the host cells to the cancer cells. Trogocytosis enables a better survey by the immune cells in the classical “immunological setting”. The failure of such mechanisms can play a role in immuno-evasion. Similarly in the “oncologic setting” trogocytosis might help cancer cells acquire new properties spontaneously or under selective pressure (initiation of metastasis, chemotherapy).

The correlation of Hospicells' infiltrate with the patients' response to neo-adjuvant chemotherapy displayed in the preliminary clinical study might lead to the prediction of spontaneous chemoresistance in patients and to optimisation of the chemotherapy regimen. One of the limitation of our TMA study is the small number of patients included, however to avoid bias linked to the heterogeneity of ovarian tumors and management protocols we have selected only patients treated by neo-adjuvant chemotherapy. Moreover chemosensitivity was rigorously defined as the absence of any active tumoral infiltrate at the time of final surgery. These findings have to be confirmed in a larger independent set of patients to raise clinical relevance.

The role of the host as major actor in controlling neoplasic disease was illustrated in several reports underlying the role of the immunologic activation in patients' prognoses. Galon et al. demonstrated that a sign of an immune response within colorectal cancers was associated with the absence of pathological evidence of early metastatic invasion and with prolonged survival [50]. These data, as well as our present data on acquired chemoresistance of ovarian cancer cells by MDR protein transfer from Hospicells, suggest that the host has a key role in the development or control of neoplastic disease. The determination of cellular mechanisms and pathways leading to intercellular recognition and “oncologic trogocytosis” with Hospicells could lead to the identification of novel therapeutic approaches targeting intercellular interactions.

## Materials and Methods

### Cell cultures

Mesothelial cells interacting with ovarian carcinoma cells were isolated from ascitis of non-previously treated patients with stage III ovarian cancer undergoing ascitis evacuation for clinical discomfort following routine protocols approved by the IRB of the Hospital Hôtel-Dieu, Paris. As ascitis evacuation is part of the routine management of patients in the medical oncology department of Hôtel-Dieu only oral consent was obtained from the patients. The ascitis were then de-identified and addressed to the research laboratory. As the laboratory and hospital have two different locations there was no way to link the ascitis to the patients' file. Five different patients' ascitis were used in this study; ascitis fluids were centrifuged at 800 rpm for 1 minute. Lymphocytes and erythrocytes were separated from cancer cell aggregates using a Ficoll procedure; dilution method was used to select ovarian cancer cells aggregated on mesothelial cells. The aggregates were separated using trypsin (Mediatech, Inc). Using serial dilution and enrichment, mesothelial cells (Hospicells) interacting specifically with the ovarian cancer aggregates were isolated and grown at 37°C in 5% CO_2_/95% air in RPMI medium (Mediatech Inc.) supplemented with 10% fetal bovine serum (FBS; Mediatech Inc.) and 5% glutamine. Human ovarian cancer cells (OVCAR3), a fibroblast cell line (CCD-976SK) and human bone marrow endothelial cells (HBMEC) were obtained from American Type Culture Collection (ATCC) and were maintained as instructed. The Tissue Micro Array were constructed from surgical specimens obtained in the Institut Claudius Regaud, the patients gave a general written informed consent and the use of these specimens was authorized by the IRB of the Institut Claudius Regaud.

### Immunohistochemistry

Stromal cells were pelleted and fixed in paraffin. Immunohistochemistry was performed on 4 µm-thick routinely processed paraffin sections. The following antibodies were used to characterize stromal cells: cytokeratin (KL1, Beckman Coulter), vimentin (V9, Beckman Coulter), CD45 (2b11 and PD7/26, Dako), CD20 (L26, Dako), CD3 (SP7, Neomarkers), CD68 (KP1 and PG-M1, Dako), CD 34 (OBend10, Dako), CD-10 (clone 56C6, Novocastra, Newcastle, UK), S100 protein (polyclonal, Dako), myeloperoxydase (polyclonal, Dako), CD166 (polyclonal, Dako), CD146 (polyclonal, Dako) and epithelial membrane antigen (E29, Dako). The antibodies were used according to the manufacturers' instructions. Antibodies against ABC proteins (QCRL1, QCRL3, MRP2, MRP3, MRP5, LRP, BXP21, and BXP34) and the anti-P-gp (CD243) antibody linked to phycoerythrin (PE) were provided by Immunotech (Marseille). To assess clinical relevance of the presence of Hospicells we retrospectively reviewed the correlation between their presence and chemoresistance. Using a tissue micro-array instrument (Beecher Instruments, Alphelys), we removed two representative areas of the tumor from paraffin-embedded tissue blocks that had been prepared at the time of resection. Tissue micro-arrays containing the tissue cores were then cut into 5-um sections for staining with Harris's hematoxylin and immunohistochemical staining. 29 patients treated by neo-adjuvant chemotherapy (e.g. six cycles of carboplatine and taxol) and secondary surgery in our cancer center between January 2000 and January 2005 were included in this preliminary study. Staging was assessed according to the International Federation of Gynecology and Obstetrics (FIGO) classification. Chemosensitivity was defined as achieving complete clinical response after completion of the first-line chemotherapy associated to a pathologic complete response defined as no evidence of disease after pathologic examination of all specimens at the time of secondary surgery. Chemoresistant was defined as patients with histologically confirmed residual disease at the time of secondary surgery. This is a more rigorous definition of resistance as compared with conventional clinical criteria. It has already been used by others and recognizes that absence of a pathologic complete response signifies the presence of a chemoresistant cellular population. Each specimen was examined for the Hospicells infiltrates within the tumor. The densities of these stromal infiltrates were scored independently (EM, AR) on a 0 to 10 scale combining a CD10 immunoblotting and morphological alaysis. All vascular-like structures were excluded from the evaluation. The clinical data were independently retrieved by another investigator (JC).

### Electron microscopy

Ovarian cancer aggregates were cultured for 48 hours. Cells were subsequently washed with the PBS and fixed for 45 min in 30% formaldehyde +5% glutaraldehyde. Thereafter, the cells were centrifuged, treated with 50 mM ammonium chlorate, dehydrated and enveloped in the Epoxy resin at low temperature at polymerization conditions. The micro sections (600–800 A°) were performed and colored with uranyl acetate and lead and visualized on a Philips CM 10 electron microscope [51]. Primary Hospicells and OVCAR3 cultures were performed and analyzed to establish the microscopic characteristics of the two different cell types and identify each one of them among the aggregates.

### Adhesion assay

Culture plates were coated with Hospicells, fibroblasts or HBMEC (Human Bone Marrow Endothelial Cells) up to 70% confluency. OVCAR3 cells overexpressing enhanced green fluorescent protein (eGFP) were added. Gentle washing after 1 hour eliminated non-adherent cells. Optical density was measured. The experiment is representative of three independent experiences.

### Matrigel cultures

Ninety six wells plates pre-coated with matrigel (Becton-Dickinson, Le Pont de Claix, France) were used and matrigel was allowed to polymerize for 1–2 hours at 37°C. Hospicells were cultivated on Matrigel for 24 hours before eGFP-Ovcar3 were seeded onto the matrigel-coated wells. Following a 24 hours incubation periods at 37°C, intercellular interactions were assessed using an inverted microscope fitted with a digital camera (Nikon-Diafot).

### Chemoresistance assay

Hospicells were first grown to 60% confluency in 96-well flat-bottomed tissue-culture plates. OVCAR3 cells over-expressing e-GFP were plated at a density of 20,000 cells/well and co-cultured for 24 hours with the hospicells before addition of 22.2 µM carboplatin or 1.4 µM paclitaxel [52]. The effect of these cytotoxic agents was assessed using a quantitative sulphorhodamine B (SRB) colorimetric assay, as described previously [53]. The same experiment was also performed using a transwell co-culture system in which Hospicells were cultured in the bottom chamber and OVCAR3 cells were cultured in the upper membrane. Both types of experiments were repeated after addition of 50 µM verapamil. To confirm the potential role of Hospicells we isolated aggregates from ascitis of non-previously treated patients with ovarian peritoneal carcinosis. We cultured these aggregates on RPMI media supplemented by calf fetal serum (10%) and antibiotics. Carboplatin and paclitaxel treatment were performed using the same conditions as in vitro. The effect of chemotherapy was assessed by conventional microscopy. The experiment is representative of three independent experiences.

### Cell transfer (“oncologic trogocytosis”) assay

The assay was carried out as previously described [54]. Cancer cells or Hospicells were stained with the lipophilic dye PKH67 (Sigma-Aldrich) or with the cytoplasmic Cell Tracker Orange CMTMR (5-(and-6)-(4-chloromethyl-benzoyl-amino-tetramethyl-rhodamine)) (Molecular Probes, Eugene, Oregon, USA) according to the manufacturer's instructions. The PKH67-labelled cells were then co-cultured for 3 hours with CMTMR-labelled cells in 96-well round-bottomed tissue-culture plates at a density of 6×10^5^ cells in 120 µl of complete RPMI 1640 medium supplemented with 10% fetal calf serum (FCS). Culture plates were centrifuged for 1 min at 700 rpm to promote cell contact and left for 1 hour at 37°C in 5% CO_2_. Cells were then washed twice with PBS containing 0.5 mM EDTA and analysed at two time-points (3 minutes and 3 hours) by flow cytometry using a LSRII cytometer and DIVA software (BD Biosciences, Mountain View, CA). Live cells were gated on the basis of forward scatter/side scatter parameters and 25,000 events were acquired in each experiment with FL1 channel (log scale) for PKH67 and FL2 channel (log scale) for CMTMR. The synaptic transfers were deducted from the PKH67 mean fluorescence of gated CMTMR-labelled cells after 3 minutes and 3 hours of co-culture. The cell transfer assay was repeated with labelled P-gp. The experiment is representative of three independent experiences.

### Confocal microscopy

PKH67-stained hospicells and CMTMR-labelled OVCAR3 cells were processed as follows. After contact for 3 minutes or 3 hours, cells were gently resuspended and plated on poly-L-lysine (Sigma-Aldrich, St Louis, MI) coated slides for 5 minutes at 37°C. After fixation with PBS containing 4% p-formaldehyde, cells were washed and directly mounted in PBS containing 90% glycerol and 2% 1-4-diazabicyclo (2.2.2) octane (DABCO, Sigma-Aldrich, St Louis, MI). Samples were examined using an LSM 410 confocal microscope (Carl Zeiss, Jena, Germany).

### Fluorescence Activated Cell Sorting (FACS)

To determine MDR protein expression in Hospicells, they were seeded at a density of 8×10^4^ cells/well. Two days later, the cells were serum-starved for 24 hours, fixed in 3% paraformaldehyde, and permeabilized with 0.1% Triton X-100 in PBS. The pattern of MDR protein expression was assessed by Fluorescence Activated Cell Sorting (FACS) using the Intraprep permeabilization reagent kit (Beckman-Coulter). Isotype controls were provided by Chemicon International Inc. (Temecula, CA, USA). Protein expression was analysed by flow cytometry (EPICS Altra, Beckman Coulter). The functionality of ABC proteins expressed by Hospicells was determined by flow cytometry using rhodamine (10^−6^ mol/L) and cyclosporine (2×10^−6^ mol/L) as probes. Their effect was expressed as a shift in the mfi of dye accumulation. For each sample, 5,000 events were collected. The experiment is representative of three independent experiences.

### Proliferation assay

96 wells plate were seeded with 20000 Hospicells. 5000 eGFP-OVCAR3 cells were then added. Culture were performed at 37°C in 5% CO_2_/95% air in RPMI medium (Mediatech Inc.) supplemented with 10% fetal bovine serum (FBS; Mediatech Inc.) and 5% glutamine. Proliferation was evaluated daily by evaluating the fluorescence of each well using a fluorescent reader (Wallac). Each condition was done in triplicate. The experiment is representative of three independent experiences.

### Statistical Analysis

Descriptive statistics were calculated for baseline demographic and clinicopathologic characteristics. Associations between CD10 immunoreactivity and clinicopathologic features were assessed by the chi-square test. For other experiments Student-t, Fisher exact or chi-square test were performed as appropriate. All p-values are two-sided with statistical significance evaluated at the 0.05 alpha level. Ninety-five percent confidence intervals (95% CI) were calculated to assess the precision of the obtained estimates. All analyses were performed in SAS Version 9.1 (SAS Institute, Inc., Cary, North Carolina) and Stata Version 8.0 (Stata Corporation, College Station, Texas). Mean±SEM are shown on the graphs.

## Supporting Information

Figure S1 Proliferative effect of Hospicells on OVCAR3 cells. In-vitro proliferation assay. Co-culture of Hospicells and eGFP-OVCAR3 cells. 96 wells plate were seeded with 20000 Hospicells. 5000 eGFP-OVCAR3 cells were then added. Culture were performed at 37°C in 5% CO2/95% air in RPMI medium supplemented with 10% Fetal Calf Serum. Proliferation was assessed daily using a fluorescent plate reader. (Representative of 3 different experiments).
(0.32 MB TIF)

Figure S2 Acquisition of Pgp by e-GFP-OVCAR3 cells when incubated with low PgP expressing Hospicells. Intercellular transfer of labelled P-gp on co-culture of Hospicells with low expression of Pgp and eGFP-OVCAR3 cells for 5 hours. As displayed there was no acquisition of PgP by eGFP-OVCAR3 cells.
(1.98 MB TIF)
