# Supplementary figures and images for: Oncologic Trogocytosis of an Original Stromal Cells Induces Chemoresistance of Ovarian Tumours
Source: PLoS One. 2008 Dec 16;3(12):e3894. doi: 10.1371/journal.pone.0003894 (PMC2597737; doi:10.1371/journal.pone.0003894)

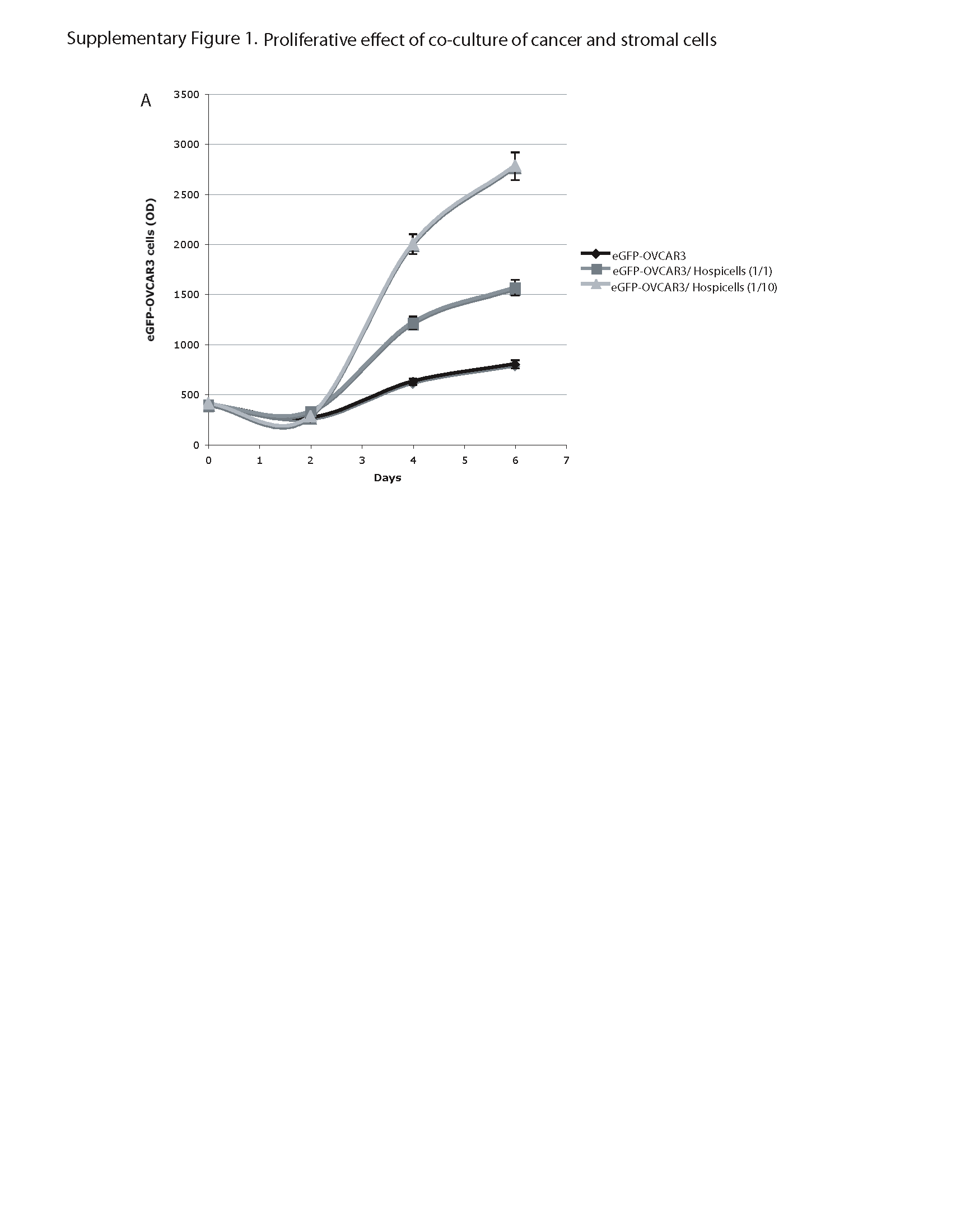

Supplement: Figure S1 — Proliferative effect of Hospicells on OVCAR3 cells. In-vitro proliferation assay. Co-culture of Hospicells and eGFP-OVCAR3 cells. 96 wells plate were seeded with 20000 Hospicells. 5000 eGFP-OVCAR3 cells were then added. Culture were performed at 37°C in 5% CO2/95% air in RPMI medium supplemented with 10% Fetal Calf Serum. Proliferation was assessed daily using a fluorescent plate reader. (Representative of 3 different experiments). (0.32 MB TIF) [file pone.0003894.s001.tif]

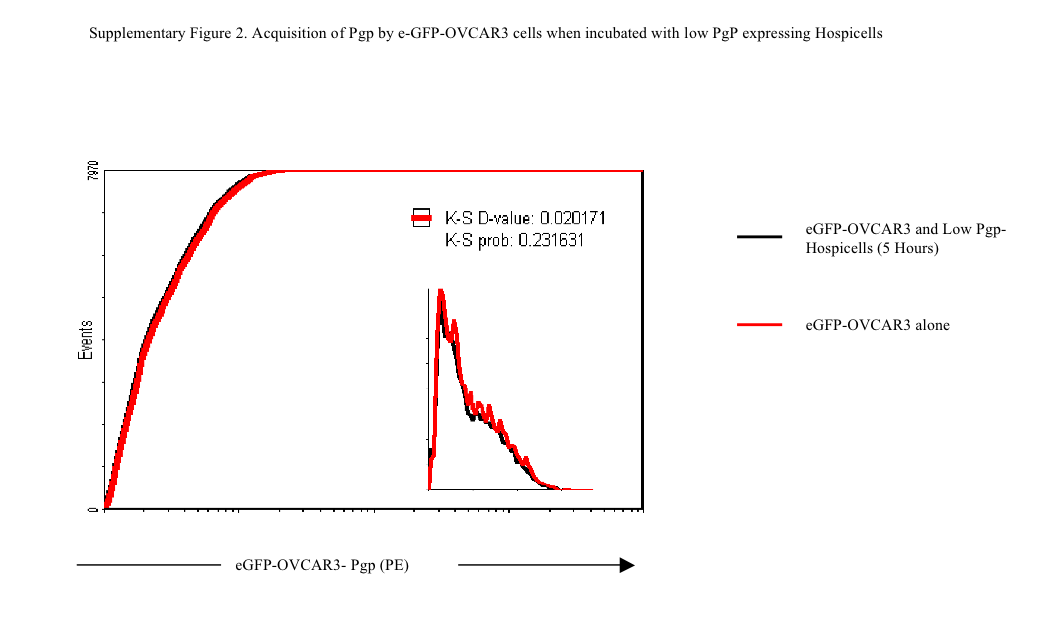

Supplement: Figure S2 — Acquisition of Pgp by e-GFP-OVCAR3 cells when incubated with low PgP expressing Hospicells. Intercellular transfer of labelled P-gp on co-culture of Hospicells with low expression of Pgp and eGFP-OVCAR3 cells for 5 hours. As displayed there was no acquisition of PgP by eGFP-OVCAR3 cells. (1.98 MB TIF) [file pone.0003894.s002.tif]
